# Professional Character

**Published:** 1866-08

**Authors:** F. S. Slosson


### PROFESSIONAL CHARACTER.

Read before the Northern Ohio Dental Association.

BY F. S. SLOSSON, D. D. S.

*Gentlemen of the Northern Ohio Dental Association:*—

It is with feelings of gratitude we acknowledge the good providence of God which has brought us through the vicissitudes of another year to this our annual meeting.

We are now to look over, criticise, and improve the past; and from this more elevated stand-point, peer into the future, that, if possible, we may see more brilliant light, and more glorious success ahead. In looking back over past years of trial, toil and doubt, we discover, perhaps, much that we could wish obscured from sight and memory. But every trial and success, yes, and every failure, too, has done something to elevate us to our present high position.

The dental profession of Northern Ohio have not been a whit behind the vanguard of any other section of our country, so far as the practical application of true principles to successful and desired results in treating teeth are sought.

Modesty might here forbid my particularizing; yet I may say that nowhere have there been earlier or more persistent and successful efforts and experiments made, than those made by the elderly members of this association, to treat, restore, and preserve the natural dental organs; but it affords us very great satisfaction to contemplate the younger members, and the facilities they now have for improvement and progress;—that where we had to creep, they may run; that where we had to grope in the dark, they may walk in meridian sunlight. But all this experience and knowledge, all this skill and these facilities will fail of the desired results, unless accompanied with a high professional and moral character. I, therefore, suggest the subject of professional character.

What, then, is character? The personal qualities of a man are his real, private, undeveloped character, naked and open only to the eyes of Him with whom we have to do. In proportion as these are good or bad, so is his real character good or bad. The development and exhibition of these qualities constitute the visible character of a man, which will be either good or bad, as these several qualities control his actions and they will inspire our confidence, and draw out our sympathies towards him; or they will incite distrust, and repel, or render formal and distant our intercourse. Hence we say, character is the representation of a man, as to personal qualities, because this is all we see or know of him.

What distinguishes one man from another, but the representation of different personal qualities? Character gives authority to the words, and importance to the decisions, of one person above those of his fellow. This fact is felt and acknowledged in all our intercourse with mankind.

That our profession should have a character as distinctly marked as that of law, medicine or divinity, will not be questioned by any one. I repeat, then, character is the representation of a man as to personal qualities. There may be associated as well as individual character.

Associated character is the combining of the personal qualities of different individuals, whether harmonious or discordant, high and refined, or low and deceptive, the development of which will give to this combination or association its true character.

If this is so, then we are to feel ourselves personally responsible for the cultivation and possession of those qualities which will elevate us as individuals, and thus elevate the profession at large.

Actions are the manifestations of character. Persistence is the quality that does most to establish it. In the language of another, character indicates the degree in which a man possesses creative, spiritual energy—is, in short, the only expression of the man—the person. The man who possesses knowledge, energy and skill, accompanied with high moral qualities, is the man who will do most for the profession. He possesses true dignity, has little of selfishness, and is above those jealousies and bickerings that disturb little minds, who are in constant fear lest some one should be benefited by coming in contact with them. He is accessible to all; and his opinions are anxiously sought, and as freely given. He secures his own by seeking the public good. His design is, that all he does shall be well done; and if he fail, as sometimes the best will do, he is ready to make it good. His charges are neither too high or too low, as either extreme destroys confidence. In short, he is governed by that higher law of doing as he would be done by. Such a character is the product of honest culture, is an honor to the profession and to mankind, and its reward is on high. But how strangely in contrast is such a character with that ignoramus, whose advent into a town is heralded by a shower of cards, bills and advertisements, found in every vehicle in our streets, in every door-yard, and under every door, proclaiming the thousands of teeth set and filled during the last few weeks, inviting the community to improve this opportunity, as they may never see his like again!

Such a man deals in base metals, soft solder, soft soap, and has a softer head. His patients pay a higher price, in proportion to value received, though he charge but two and sixpence per plug, and a bit or a picayune for extracting; he being a bit of a picayune man, the perfume of his soap is exceedingly attractive with a certain portion of community whose ideas assimilate his own. Such a man is the product of the enemy's sowing, a disgrace to the profession and his kind;—whose end is to be burned. Between these two men, we have every shade and grade of character. We are glad to know the number who approximate the first is daily increasing. Yet it is lamentably true that our profession is clogged in its upward flight by a multitude who are nearly assimilated to the latter.

I said that actions are the manifestations of character. It is not one or two great, good or noble acts that make or establish the character of a man. It is formed only by numberless little acts. As dust shows the way the wind blows, so do the little acts of a man indicate more truly his real character than great ones. One little act does but little, seemingly, to establish a good character; yet it does very much in destroying it.

How quickly do we form an opinion of a man from his advertisements! Is it not that they are so unlike those of other professions? We are prone to ride a hobby of some kind, and the personal pronoun is the motive power; as, for instance, "I extract teeth with a peculiar kind of an instrument without pain." "I destroy the nerve without pain." "I plug over exposed nerves." "I hammer in my plugs with a mallet." "I put in porcelain plate, and I do all kinds of work."

To all this is added the testimony and recommendation of some professor, clergyman, lawyer, or surgeon. Now all this savors of quackery; and the longer and more numerous the recommendations, the greater the humbug. The fact is, if we are riding a hobby of any kind, it will be likely to appear in our advertisements, with head and tail up.

Character is the embodiment of the acts of a man. Persistence is that quality that does most to establish it. It is the stream that has longest run that has made the deepest cut in the mountain rock. So it is with this quality. It is the persistence, the tenacity which holds a man to his work, that makes the strongest mark upon him.

How often do we see men of the most brilliant minds, and fertile imaginations, fail to make or establish a character that does not expire with their breath; and for the reason they do not possess this quality to any considerable extent. While, on the other hand, we see men of moderate attainments, and less brilliant minds, engraving their names and their characters on a nation's heart, and in a nation's history.

Persistence in a good enterprise inspires confidence. It shows the man in his true character, that he is tenacious of his principles, and proud of his profession; while his opposite is all things to all men, and of little account to any one.

This quality is the more effective for evil than for good, because of the downward tendencies of our nature. It is persistence in an evil course that has furnished the victims for our prisons and the scaffold, forcing, yearly, thousands of intemperate men down to drunkard's graves.

I have dwelt the more on this quality because of its importance, and of the results which might be accomplished, if each individual in the profession would persistently pursue such a course as would fit him for his work, and make him an honor to his profession. Let a young man determine that on the next tooth he would spend all the time and care necessary to excavate and prepare it for the reception of the gold, then introduce slowly, and condense thoroughly, until it could not be punctured with the point of a sharp instrument, then burnish and polish until he could say, I have done the best I could, and thus proceed with the next and the next, holding to his determination, would it be long before he would stand in the front rank of the profession in this department?

There are three things necessary to the attainment of a good professional character, viz: an object to be aimed at, the means by which it is to be attained, and persistence in the use of those means.

If a young man aims to be first in his profession, his object is a noble one, and is attainable, if he improve his time, and the means within his reach, (and they are a thousand fold more now than when some of us commenced,)—if he tenaciously hold to his determination, and to his work, until it becomes the object of his highest aspirations. As another has said, "The true merchant, the true statesman, the true military commander, becomes a man of character only when he puts on and identifies himself with his particular profession or art."

The want of this application, and this personal identification with the object in pursuit, is a reason why so many accomplish so little, and rise no higher in the profession. It is also the reason why so many resort to those mean and deceptive practices which are a perfect infliction on the community, such as extracting teeth because difficult to plug; plugging cavities that are accessible, and leaving those that are not; taking out plugs which are preserving the teeth, for the sake of replacing them; putting in plugs of gold that are two-thirds tin; putting in plates of gold which are such only in appearance. These are little things; but they are things we have often seen, and they as strongly indicate character as larger ones. Any such resort shows the man to be wanting in one essential quality of a good professional character, viz: self-respect. Inasmuch as his design is to get from his brother, in this low way, that which he might have by honorable procedure. This is taking a dollar and cent view of the whole practice. The aim is low, and the practice lower.

When a patient takes our chair, he commits to us a personal trust, not of dollars and cents, but a trust infinitely more sacred; and any person who does not feel its responsibility, and is recreant of this trust, is unfit for his position, and unworthy of a standing in our profession. "Brethren, we are persuaded better things of you, though we thus speak."

No man has attained to eminence as a military man, lawyer or physician, but by long years of toil, trial, and embarassment in the primary principles of his profession. Every step in his progress has been attended by unforeseen difficulties, which he has mastered, and thus, step by step, has he followed his own aspirations to the summit of human glory.

While another one, surrounded by circumstances equally, or more advantageous, whose aim is low, has hardly a respectable standing in his profession. Why is this? The one relies on his own native energy, upon improving every means in his power, learning something from every one; while the other relies more on the accidental circumstances which surround him, or on some title of D. D. S., M. D., or D. D. Esq. All of which are good and gratifying, and always retiring when worthily received. But these are his advertisements—they stand out in bold relief and are the first things seen, and his capital. They are also the true indications of his character.

It is said of Daniel Webster, that on receiving his diploma, he retired, and in the presence of his classmates tore it in pieces. Whether this is true or not, it is eminently true that he relied upon his own native energy—that he laid with his own hands every stone in that foundation on which was to be built the character of that illustrious statesman.

The dental profession is in its infancy. Its members have been in years past like men deep down in some dark mine—each one working and carrying his light on his own head, borrowing none and reflecting none. But I rejoice to see an elevating power—a germ of progress, the adoption of those principles which are to place the profession in a high position of usefulness, dispersing the gloom of that narrow-minded selfishness, which dries up and shuts up all the hidden and nobler qualities of the soul.

The dental colleges and periodicals, the dental associations and conventions, the social gatherings, and free interchange of views of practice and kindly feeling, all speak well for progress in dental science, and serves to augment the stream of blessings which shall add immeasurably to the happiness and duration of human life, as it flows on to ages yet to come.

There are now facilities for improvement of which a few years since we knew nothing. The veriest sprig in the profession feels its influence, and straitens up himself to be more of a man.

It makes us wish to forget the past, and begin again. But as without the past there could be no future, and as every elevation must have some means of ascent, we will rejoice if the past be made the stepping stone by which any shall rise to higher attainments, or to a more lofty eminence.

Brethren, in leaving the chair, I thank you for the expression of your confidence in placing me in it; and hope the same harmony and good feeling may characterize all our future meetings.

